# Clinical efficacy and safety of combined anti-BCMA and anti-CD19 CAR-T cell therapy for relapsed/refractory multiple myeloma: a systematic review and meta-analysis

**DOI:** 10.3389/fonc.2024.1355643

**Published:** 2024-04-08

**Authors:** Han Xu, Chaoyang Guan, Peipei Xu, Dongming Zhou, Yong Xu, Bing Chen, Hua Bai

**Affiliations:** ^1^ Department of Hematology, Nanjing Drum Tower Hospital, Affiliated Hospital of Medical School, Nanjing University, Nanjing, China; ^2^ School of Medicine, Southeast University, Nanjing, China

**Keywords:** CAR-T, BCMA, CD19, myeloma, efficacy, safety, meta-analysis

## Abstract

**Background:**

The low rates of durable response against relapsed/refractory multiple myeloma (RRMM) in recent studies prompt that chimeric antigen receptor (CAR)-T cell therapies are yet to be optimized. The combined anti-BCMA and anti-CD19 CAR-T cell therapy showed high clinical efficacy in several clinical trials for RRMM. We here conducted a meta-analysis to confirm its efficacy and safety.

**Methods:**

We collected data from Embase, Web of Science, PubMed, CNKI, Wanfang and Cochrane databases up to April 2023. We extracted and evaluated data related to the efficacy and safety of combined anti-BCMA and anti-CD19 CAR-T cell therapies in RRMM patients. The data was then analyzed using RevMan5.4 and StataSE-64 software. PROSPERO number was CRD42023455002.

**Results:**

Our meta-analysis included 12 relevant clinical trials involving 347 RRMM patients who were treated with combined anti-BCMA and anti-CD19 CAR-T cell therapies. For efficacy assessment, the pooled overall response rate (ORR) was 94% (95% CI: 91%-98%), the complete response rate (CRR) was 50% (95% CI: 29%-71%), and the minimal residual disease (MRD) negativity rate within responders was 73% (95% CI: 66%-80%). In terms of safety, the pooled all-grade cytokine release syndrome (CRS) rate was 98% (95% CI: 97%-100%), grade≥3 CRS rate was 9% (95% CI: 4%-14%), and the incidence of neurotoxicity was 8% (95% CI: 4%-11%). Of hematologic toxicity, neutropenia was 82% (95% CI: 75%-89%), anemia was 71% (95% CI: 53%-90%), thrombocytopenia was 67% (95% CI: 40%-93%) and infection was 42% (95% CI: 9%-76%). The median progression-free survival (PFS) was 12.97 months (95% CI: 6.02-19.91), and the median overall survival (OS) was 26.63 months (95% CI: 8.14-45.11).

**Conclusions:**

As a novel immunotherapy strategy with great potential, the combined anti-BCMA and anti-CD19 CAR-T cell therapy showed high efficacy in RRMM, but its safety needs further improvement. This meta-analysis suggests possible optimization of combined CAR-T therapy.

**Systematic review registration:**

https://www.crd.york.ac.uk/PROSPERO/, identifier CRD42023455002.

## Introduction

Multiple myeloma (MM) is the second most prevalent hematological malignancy (1). Despite significant improvement in treatment outcomes due to novel agents, such as immunomodulatory drugs, protease inhibitors and anti-CD38 monoclonal antibodies, the prognosis remains grim for refractory/relapsed multiple myeloma (RRMM) (2, 3). Currently, anti-BCMA chimeric antigen receptor (CAR)-T cell therapies have yielded significant clinical outcomes in RRMM patients, with anti-BCMA CAR-T cell products approved by the US FDA (idecabtagene vicleucel in March 2021 and ciltacabtagene autoleucel in February 2022 (4, 5)), that Phase II clinical trials demonstrated overall response rates (ORR) of 70%-95% (6, 7). However, due to the high relapse rate of post-CAR-T cell therapies, largely attributed to antigen escape (8), there is a need for more effective CAR-T-based regimens.

CD19, which is expressed on multiple differentiated MM cells and myeloma-like stem cells, is associated with enhanced MM drug resistance and tumor-propagation (9). Previous studies have reported the synergistic effects of CD19-targeted therapies with other MM therapies (10). The combination of anti-CD19 CAR-T cell therapy and autologous stem cell transplantation has shown significant clinical outcomes in RRMM patients (11). Additionally, the ultra-low expression of CD19 in MM cells can trigger elimination by anti-CD19 CAR-T cell therapies (12), establishing CD19 as a crucial biomarker of RRMM and a therapeutic target of CAR-T cell therapies. Multiple studies utilizing anti-BCMA and anti-CD19 CAR-T infusion have obtained significant clinical outcomes in proof-of-concept trials for RRMM patients (13–15).

This meta-analysis discusses the clinical efficacy and safety of combined anti-BCMA and anti-CD19 CAR-T cell therapies in RRMM. The strengths of this study include the large sample size, comprehensive range of clinical trials, valuable evaluation index system and in-depth subgroup analyses, collectively facilitating the application of combined infusion with CAR-T cell therapies.

## Materials and methods

### Methods

This pre-registered systematic review with meta-analysis adheres to the guidelines provided by the PRISMA checklist (PROSPERO reference number # CRD42023455002) (16).

### Literature search

We conducted a comprehensive search across various databases including Embase, PubMed, CNKI, Wanfang Databases, Cochrane library, and Web of Science. Records were searched from their inception up until April 20, 2023, without any language restrictions. Our search strategy employed a combination of free-text terms and Mesh terms related to “BCMA”, “CD19”, “chimeric antigen receptor”, “CAR” and “multiple myeloma”. To ensure a thorough approach, a comprehensive search of PROSPERO for relevant systematic reviews was also performed.

### Selection criteria

Inclusion criteria for the studies were as follows: study types were Phase I and II clinical trials or retrospective analyses, whether single center or multicenter; the inclusion of patients with RRMM; treatment of patients with combined anti-BCMA and anti-CD19 CAR-T cell therapies; and reporting of outcomes data such as partial response (PR), very good partial response (VGPR), complete response (CR), overall response rate (ORR), minimal residual disease (MRD) negativity, overall survival (OS), progression-free survival (PFS), cytokine-release syndrome (CRS), neurotoxicity, hematologic toxicity (neutropenia, anemia and thrombocytopenia), infection and recurrence. Studies were excluded if they did not meet the following criteria: less than 5 patients participating in the clinical study; treatment of patients with combinations of other therapies; or if they were case reports, observational studies, animal studies, reviews or abstracts.

### Data extraction

We defined the inclusion criteria according to the PICOS criteria. P: participants (RRMM patients), I: interventions (combined anti-BCMA and anti-CD19 CAR-T cell therapy), C: comparisons (this analysis included single-arm studies without a control group), O: outcomes (the outcome was the efficacy and safety of combined anti-BCMA and anti-CD19 CAR-T cell therapy), S: study designs (Phase I and II clinical trials or retrospective analyses). Data from studies meeting the inclusion criteria were independently and dually extracted by two authors of this study. The extracted features include study characteristics (author, publication time and median follow-up time), patient characteristics (number, age, gender, high-risk cytogenetics, the proportion of ISS III stage, extramedullary disease, prior lines of therapy, time since diagnosis, mAb exposed, prior ASCT, BCMA and CD19 positivity requirement at enrollment), characteristics of combined anti-BCMA and anti-CD19 CAR-T cell therapy (infusion dose, infusion time, conditioning treatment, antigen-recognition domain, costimulatory molecule and loading regimen) and study outcomes (clinical response, MRD negativity rate, recurrence rate and toxicity). In the cases of disagreement between the two authors, a third person in this study settled the discussion.

### Literature quality assessment and publication bias

The Methodological Index for Non-randomized Studies (MINORS) (17) was employed to assess the quality of the incorporated studies. The index comprises 12 points, with four specifically designed for comparative research. Only the first eight items were used to evaluate the included studies, as none of them had a control group. The maximum score was 16 points for each study, with scores ranging from 0 to 2 for each item. We used Egger test, Begg test and funnel plots to explore the potential publication bias.

### Statistical analysis

All statistical analyses were conducted using RevMan5.4 and StataSE-64 software. We applied the inverse variance method to consolidate the effect estimates. To derive pooled results from various studies, we calculated both response rates and rates of adverse effects, providing them with their respective 95% confidence intervals (CI). The chi-squared test and the I-squared test (I^2^ test) were used for assessing the heterogeneity among the included studies. The fixed effect model was applied when I^2^<50%, indicating that heterogeneity was not statistically significant while the random effect model was employed when I^2^≥50% suggesting that there was heterogeneity in the literature. The heterogeneity was further investigated by organizing subgroups based on age (<55 and ≥55 years), gender (male≥50% and male<50%), infusion dose (high dose group≥500×10^6^ cells or 10×10^6^ cells/kg and low dose group<500×10^6^ cells or 10×10^6^ cells/kg), infusion time (same day and not same day), antigen-recognition domain origin (Murine and Human), costimulatory molecule (4-1BB and others), the median time from diagnosis (<2 and ≥2 years), lines of prior treatment (<4 and ≥4), previous ASCT (<50% and ≥50%), high-risk cytogenetics (<50% and ≥50%), extramedullary disease (<20% and ≥20%), the proportion of ISS III stage (<50% and ≥50%), mAb exposed (<20% and ≥20%), and conditioning treatment (Cy+Flu and Cy+Busulfan). We defined a *p* of ≤0.05 as statistically significant. We also conducted a sensitivity analysis to identify and remove potentially high-risk bias studies, and to determine whether these studies significantly affect the outcomes.

## Results

### Literature search results and characteristics

**Figure 1:**
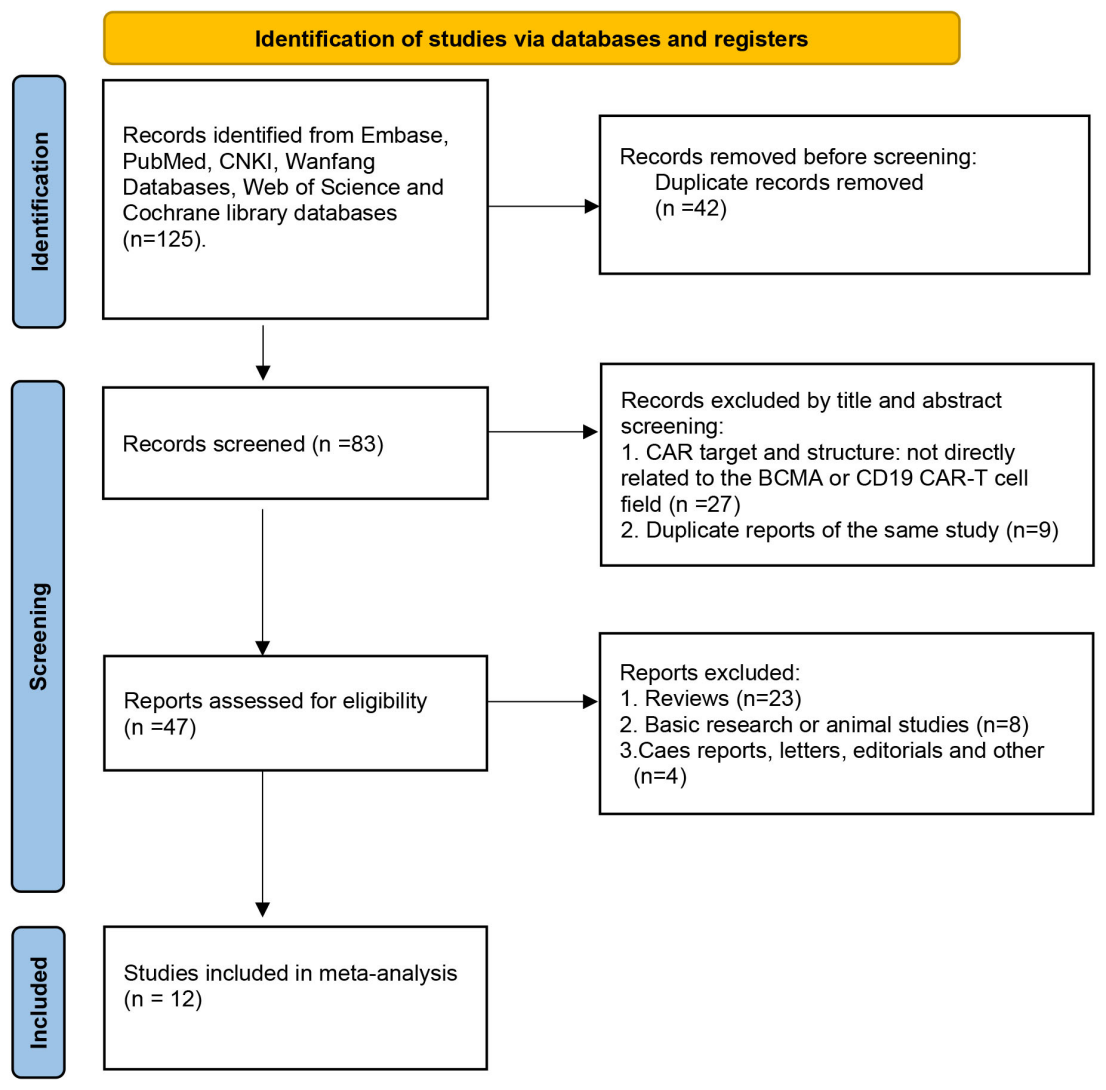
PRISMA flow diagram for the record selection process.

The PRISMA flow diagram illustrates the search strategy used to locate relevant literature (**Figure 1**). A comprehensive database search yielded 125 records, 42 of which were duplicates and were eliminated. An additional 36 records were excluded based on their title/abstract information. The remaining 47 potential pieces of relevant literature underwent further review. Following a more detailed assessment, 12 studies involving 347 participants were selected for meta-analysis (13–15, 18–26). **Tables 1**, **2** provide a summary of the characteristics of the 12 studies and the 347 patients treated with combined anti-BCMA and anti-CD19 CAR-T cell therapies, respectively.

**Table 1:** Characteristics of included studies.

NO.	First author	Year	n=	Median Follow-up Time (month)	Median PFS (month)	Median OS (month)	Conditioning treatment	CAR-T infusion dose	CAR-T infusion time	Antigen-recognition domain	Costimulatory molecule	Loading	T cell origin	Clinical response	Toxicity	MRD negativity rate	Recurrence rate within 1 year (%)	Recurrence rate within 2 years (%)	MINORS score
1	Alfred L. Garfall	2019	10	5	4.5+	—	Flu 30 mg/m2+ Cy 300 mg/m2 daily for 3d	BCMA: 5×10^8 cells CD19: 5×10^8 cells	Infused on the same day (Day1: 10%, Day2: 30% Day3 60%)	—	—	—	—	ORR:0.8 sCR+CR:0.1 VGPR:0.4 PR:0.3 SD:0.2	CRS gr. 1–2:0.8	—	—	—	12
2	Alfred L. Garfall	2023	20	30+	7.85	26.6+	Flu 30 mg/m2+ Cy 300 mg/m2 daily for 3d	BCMA: 5×10^8 cells CD19: 5×10^8 cells	Infused on the same day (Day1: 10%, Day2: 30% Day3 60%) CART-BCMA was infused first and CART-CD19 infused after at least 1 hour of observation	A fully human anti-BCMA scFv and a fully human anti-CD19 scFv.	4-1BB	Lentiviral	—	ORR:0.95 sCR+CR:0.25 VGPR:0.4 PR:0.3 MR:0.05	CRS gr. 1–2:0.85 Neurotoxicity:0.05 Neutropenia:0.75 Anaemia:0.25 Thrombocytopenia:0.3 Infection:0.15	0.65	0.55	0.8	16
3	Dian Zhou	2023	35	20.5	—	—	Flu 30 mg/m2 on Day -5 to -2 + Cy 750 mg/m2 on Day -5	—	—	—	—	—	Autologous	ORR:0.66	CRS gr. 1–2:0.83 gr. 3–4:0.17 Infection:0.457	—	0.343		14
4	Hujun Li	2022	54	24.3	16.4	42.8	Flu 30 mg/m2 on Day-5 to -3+ Cy 750 mg/m2 on Day-5	BCMA: 1× 10^6 cells/kg CD19: 1×10^6 cells/kg	Infused on the same day (on Day 0)	A murine anti-BCMA scFv and a fully human anti-CD19 scFv.	4-1BB, CD3ζ	—	Autologous	ORR:0.94 sCR+CR:0.53 VGPR:0.22 PR:0.19 SD:0.06	CRS gr. 1–2:0.87 gr. 3–4:0.13 Infection:0.48 Neutropenia:0.17 Severe anemia:0.46 Thrombocytopenia:0.61 Neurotoxicity:0.04	—	0.62		14
5	Lingzhi Yan	2019	28	16	8	16	Flu + Cy	BCMA: 2-6.8×10^7cells/kg CD19: 1×10^7cells/kg	CD19: Infused on Day 0 BCMA: 40% on Day1, 60% on Day2	A fully human anti-BCMA scFv and a fully human anti-CD19 scFv.	BCMA:CD28, CD3ζ CD19: CD28, CD3ζ, IL-6 shRNA cassette	Lentiviral	—	ORR:0.93 sCR+CR:0.41 VGPR:0.3 PR:0.19 MR:0.04 SD:0.04	CRS gr. 1–2:0.68 gr. 3–4:0.32 Severe anemia:0.28 Anaemia:1 Cytopenia:1	—	0.04	0.14	14
6	Lingzhi Yan	2020	10	20+	8+	—	Flu 30 mg/m2+ Cy 300 mg/m2 daily on Day-5 to -3	BCMA: 3.0, 5.0 and 6.5 ×10^7cells/kg CD19: 1×10^7cells/kg	CD19: Infused on Day 0 BCMA: 40% on Day1, 60% on Day2	A fully human anti-BCMA scFv and a fully human anti-CD19 scFv.	BCMA:CD28, CD3ζ CD19: CD28, CD3ζ, IL-6 shRNA cassette	Lentiviral	70%: Autologous 30%: Allogenic	ORR:0.9 sCR:0.4 PR:0.5	CRS gr. 1–2:0.9 gr. 3–4:0.1 Neurotoxicity:0.1 Neutropenia:0.9 Anemia:0.9 Thrombocytopenia:0.8 Infection:0.4 Fever:0.9	—	0.7	0.7	16
7	Sha Ma	2023	56	—	—	—	Flu 30 mg/m2 on Day-5 to -3+ Cy 750 mg/m2 on Day-5	BCMA: 2.0×10^6 cells/kg CD19: 2.0×10^6 cells/kg	Infused on the same day (on Day 0)	A murine anti-BCMA scFv and a human anti-CD19 scFv.	4-1BB, CD3ζ	Lentiviral	—	—	CRS gr. 1–2:0.86 gr. 3-4:0.04 Neurotoxicity:0.16	—	—	—	8
8	Xiaolan Shi	2018	9	3	—	—	Busulfan+ Cy followed by infusion of autologous stem cells	CD19: 1×107 cells/kg BCMA: —	CD19: Infused on Day 0 BCMA: 40% on Day1, 60% on Day2	—	CD28, OX40, CD3ζ	Lentiviral	Autologous	ORR:1 sCR+CR:0.33 VGPR:0.67	CRS gr. 1–2 :1 Fatigue:1 Infection:1 Coagulation disorders:0.67	0.67	—	—	12
9	Xiaolan Shi	2019	32	13	10.7	21.1	Busulfan+ Cy or Melphalan followed by infusion of autologous stem cells	CD19: 1×107/kg(on d0) BCMA: —	CD19: Infused on Day 0 BCMA: 40% on Day1, 60% on Day2	—	CD28, OX40, CD3ζ	Lentiviral	Autologous	ORR:1 sCR+CR:0.72	CRS gr.1-2:0.97 gr.3-4:0.03	0.64	0.16	—	14
10	Xiaolan Shi	2021	10	42	30+	—	Busulfan 2.4 mg/kg daily on Day-8 to -5+ Cy 1.8 g/m2 on Day-4 to -3	BCMA: 5.0×10^7cells/kg CD19: 1.0×10^7cells/kg	CD19: Infused on Day 0 BCMA: 40% on Day1, 60% on Day2	A murine anti-BCMA scFv and a murine anti-CD19 scFv.	BCMA: CD28, OX40, CD3ζ CD19: 4-1BB, CD3ζ	Lentiviral	Autologous	ORR:1 sCR:0.9 CR:0.1	CRS gr. 1–2 :1 Thrombocytopenia:1 Anaemia:1 Neutropenia:0.9 Infection:1	0.7	0	0.2	12
11	Ying Wang	2022	62	21.3	18.3	—	Flu 30 mg/m2 on Day-5 to -3+ Cy 750 mg/m2 on Day-5	BCMA: 1.0×10^6 cells/kg CD19: 1.0×10^6 cells/kg	Infused on the same day (on Day 0)	—	BCMA: 4-1BB CD3ζ CD19: 4-1BB CD3ζ	Lentiviral	—	ORR:0.92 sCR+CR:0.59 VGPR:0.19 PR:0.13 MR:0.02 SD:0.06	CRS gr. 1–2:0.85 gr. 3–4:0.1 Neurotoxicity:0.11 Infection:0.41 B-cell dysplasia:0.3 Cytopenia:0.17 Hypogammaglobulinemia:0.3	0.77	0.46		16
12	Zhiling Yan	2019	8	—	—	—	Flu 30 mg/m2 on Day-5 to -3 + Cy 750 mg/m2 on Day-5	BCMA: 1.0×10^6 cells/kg CD19: 1.0×10^6 cells/kg	Infused on the same day (on Day 0)	A murine anti-BCMA ScFv and a human anti-CD19 scFv.	4-1BB, CD3ζ	Lentiviral	—	ORR:0.95 sCR+CR:0.57 VGPR:0.24 PR:0.14	CRS gr. 1–2:0.86 gr. 3–4:0.04 Neutropenia:0.86 Anemia:0.62 Thrombocytopenia:0.62 Neurotoxicity:0.095 Infection:0.05 Fever: 0.90	0.81	0.05	—	14

PFS, progression-free survival; OS, overall survival; Cy, cyclophosphamide; Flu, fludarabine; ScFv, single-chain variable fragment; ORR, overall response rate; sCR, stringent complete response; CR, complete response; MRD, minimal residual disease; CRS, cytokine release syndrome; gr, grade.

**Table 2:** Characteristics of included patients.

NO.	First author	Year	Median age	n=	Male/Female	ISS3 (%)	High-risk cytogenetics (%)	Extramedullary Disease (%)	Prior lines of therapy (median)	Time since diagnosis (years)	mAb exposed (%)	Prior ASCT (%)	BCMA and CD19 positivity requirement at enrollment (%)
1	Alfred L. Garfall	2019	55.7	10	—	90%	—	—	3.6	—	—	—	—
2	Alfred L. Garfall	2023	57.5	20	6/14	35%	75%	—	2.25	1.95	—	40%	—
3	Dian Zhou	2023	57	35	20/15	54.30%	—	28%	4	1.92	28.60%	28%	—
4	Hujun Li	2022	58	54	26/32	44%	28%	28%	4	3.33	—	28%	—
5	Lingzhi Yan	2019	57.5	28	23/5	—	—	—	3	—	—	—	—
6	Lingzhi Yan	2020	56.3	10	7/3	—	60%	20%	3.8	—	20%	60%	BCMA:80.6%
7	Sha Ma	2023	57	56	—	—	—	—	—	—	25%	—	—
8	Xiaolan Shi	2018	56	9	8/1	33%	—	—	—	—		100%	BCMA: 81.7%
9	Xiaolan Shi	2019	53	32	8/24	—	—	—	—	—	3.10%	100%	—
10	Xiaolan Shi	2021	54	10	7/3	40%	80%	10%	—	—		100%	CD19: 0 BCMA: 50.3%
11	Ying Wang	2022	58	62	34/28	48%	29%	24%	4	2.5	32.30%	27%	—
12	Zhiling Yan	2019	58	21	10/11	19%	24%	—	6	—	5%	14%	—

ISS3, phase III in the International Staging System; mAb, monoclonal antibody; ASCT, autologous stem cell transplant.

### Quality assessment

**Figure 2:**
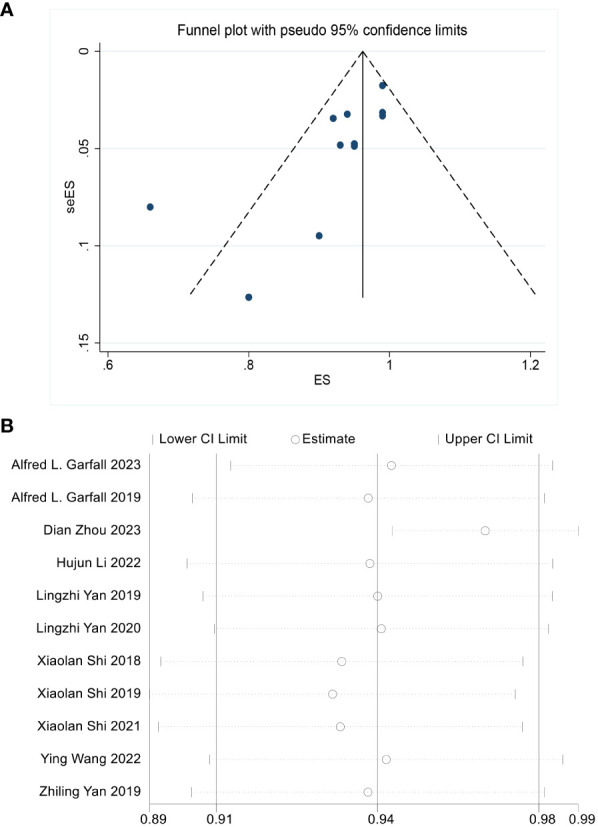
**(A)** No significant publication bias is noted on the funnel plot. **(B)** Sensitivity analysis is performed by the “Leave-One-Out” approach to assess the stability of our results, the scatter plot shows the result is stable.

The median MINORS score for the 12 noncomparative studies was 14 (range 8-16). Quality assessment results showed the eligible quality of the included studies (**Table 3**). The robust and consistent results of this meta-analysis were evidenced in the funnel diagram, showing no sign of potential publication bias for the overall response (**Figure 2A**). The ORR sensitivity analysis indicated that the effect size of the outcome index remained the same regardless of the elimination of any of the studies (**Figure 2B**).

**Table 3:** The scores of MINORS.

First author	Year	Clearly stated aim	Inclusion of consecutive patients	Prospective collection of data	Endpoints appropriate to the aim of the study	Unbiased assessment of the study endpoint	Follow-up period appropriate to the aim of the study	Loss to follow up less than 5%	Prospective calculation of the study size	MINORS score
Alfred L. Garfall	2019	2	1	1	2	1	1	2	2	12
Alfred L. Garfall	2023	2	2	2	2	2	2	2	2	16
Dian Zhou	2023	2	2	1	2	2	2	2	1	14
Hujun Li	2022	2	1	2	2	1	2	2	2	14
Lingzhi Yan	2019	2	2	2	2	0	2	2	2	14
Lingzhi Yan	2020	2	2	2	2	2	2	2	2	16
Sha Ma	2023	2	0	1	2	0	1	1	1	8
Xiaolan Shi	2018	2	0	2	2	0	2	2	2	12
Xiaolan Shi	2019	2	2	1	2	2	2	2	1	14
Xiaolan Shi	2021	2	0	2	2	0	2	2	2	12
Ying Wang	2022	2	2	2	2	2	2	2	2	16
Zhiling Yan	2019	2	2	2	2	1	2	1	2	14

### Effectiveness outcomes from meta-analysis

**Figure 3:**
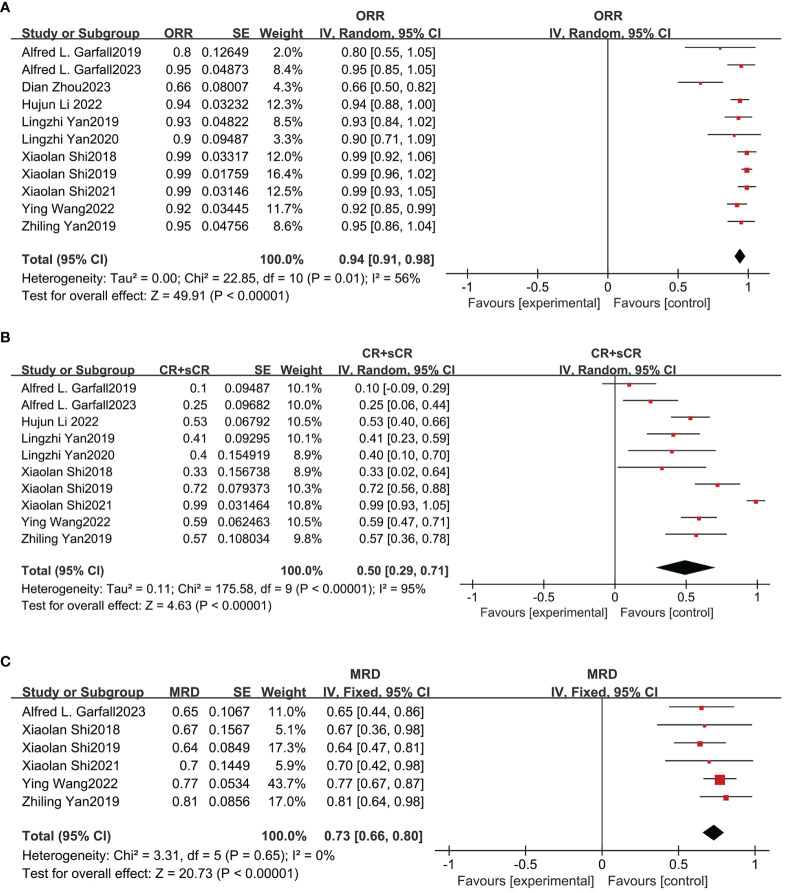
Pooled rates of OR, CR and MRD negativity in patients. **(A)** The pooled ORR is 94%. **(B)** The pooled CRR is 50%. **(C)** The pooled rate of MRD negativity is 73%.

The effectiveness of combined anti-BCMA and anti-CD19 CAR-T cell therapy in RRMM patients was based on 12 studies, yielding favorable outcome rates. The pooled ORR was 94% (95% CI: 91%-98%; **Figure 3A**) according to data from 11 studies involving 291 patients. In ten studies reporting the CRR, the pooled CRR was 50% (95% CI: 29%-71%; **Figure 3B**) among 256 patients. The pooled MRD negativity rate was 73% (95% CI: 66%-80%; **Figure 3C**) among six trials that assessed the MRD. The pooled sCR, CR, VGPR, and PR were 42% (95% CI: 24%-61%; **Supplementary Figure 1A**), 16% (95% CI: 8%-24%; **Supplementary Figure 1B**), 29% (95% CI: 20%-39%; **Supplementary Figure 1C**) and 18% (95% CI: 13%-23%; **Supplementary Figure 1D**), respectively. The median PFS was 12.97 months (95% CI: 6.02-19.91), and the median OS was 26.63 months (95% CI: 8.14-45.11).

### Safety outcomes from meta-analysis

**Figure 4:**
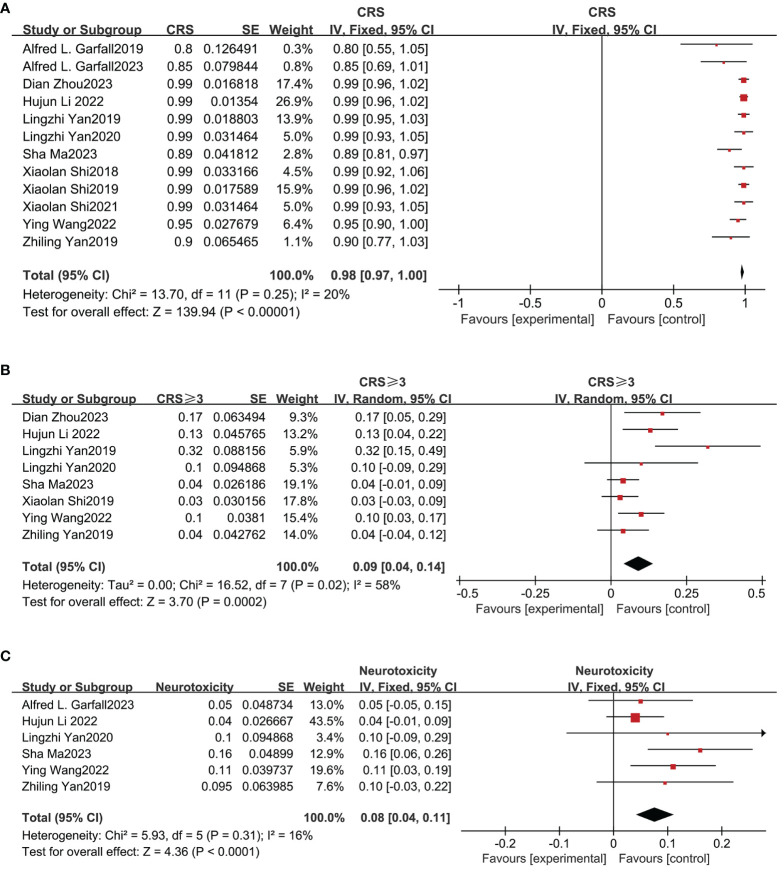
Pooled incidence of all grade CRS, grade≥3 CRS and neurotoxicity. **(A)** The pooled incidence of all grade CRS is 98%. **(B)** The pooled incidence of grade≥3 CRS is 9%. **(C)** The pooled incidence of neurotoxicity is 8%.

The safety of combined anti-BCMA and anti-CD19 CAR-T cell therapy in RRMM was also evaluated in our meta-analysis. CRS was the most common adverse event (AE). **Figure 4A** shows that the total incidence of any grade CRS was 98% (95% CI: 97%-100%) among 12 studies; and **Figure 4B** shows the pooled incidence of grade≥3 CRS was 9% (95% CI: 4%-14%). Based on the six studies that reported neurotoxicity, the relevant pooled incidence of neurotoxicity was 8% (95% CI: 4%-11%; **Figure 4C**). Hematologic toxicity was the most frequent therapy-related AE of grade≥3, including neutropenia (82%, 95% CI: 75%-89), anemia (71%, 95% CI: 53%-90%), and thrombocytopenia (67%, 95% CI: 40%-93%) (**Supplementary Figure 2**). The pooled incidence of infection was 42% (95% CI: 9%-76%; **Supplementary Figure 3**).

### Recurrence outcomes from meta-analysis

**Figure 5:**
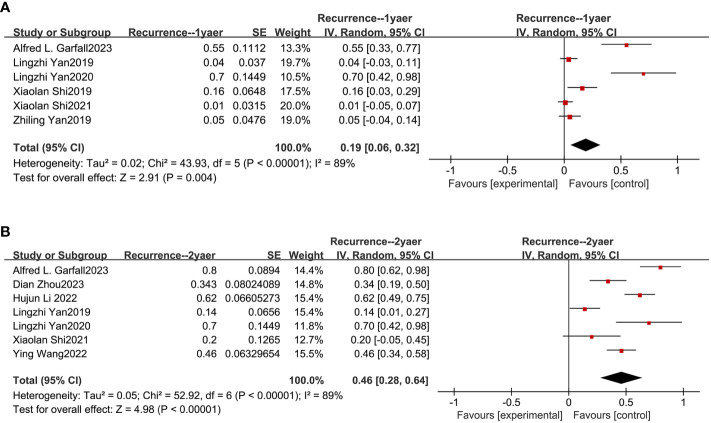
Pooled rates of recurrence within 1 and 2 years. **(A)** The pooled rate of recurrence within 1 year is 19%. **(B)** The pooled rate of recurrence within 2 years is 46%.

Based on nine studies, our meta-analysis also evaluated the recurrence rates of combined anti-BCMA and anti-CD19 CAR-T cell therapies in RRMM (13–15, 18, 20, 21, 23, 24, 26). The combined recurrence rate within one year was 19% (95% CI: 6%-32%; **Figure 5A**) and two years was 46% (95% CI: 28%-64%; **Figure 5B**).

### Subgroup analysis outcomes of meta-analysis

Subgroup analysis was utilized to investigate factors potentially influencing the effectiveness and safety outcomes of combined anti-BCMA and anti-CD19 CAR-T cell therapy in RRMM patients (**Table 4**). The factors analyzed include age, gender, infusion dose, infusion time, CAR structures, median time from diagnosis, lines of prior treatment, prior ASCT (%), ISS III level (%), high-risk cytogenetics (%), extramedullary disease (%), mAb exposed (%), and conditioning treatment. The ORR subgroup analysis demonstrated that individuals with improved illness status had a relatively higher ORR than other participants. More specifically, patients under 55 years displayed superior ORR compared to patients of ≥55 years (99% vs. 92%, *p*=0.02). Additionally, participants with ISS I/II stages of disease reported significantly higher ORR than those at the ISS III stage (96% vs. 70%, *p*=0.0002). Compared to the participants who received prior ASCT≥50%, a significantly higher ORR was attained with those who received prior ASCT<50% (99% vs. 91%, *p*=0.03). A substantially higher ORR was obtained in participants without extramedullary disease compared to those suffering from it (99% vs. 87%, *p*=0.05). Participants whose conditioning treatment was Cy+Busulfan had a higher ORR than those whose conditioning treatment was Cy+Flu ((99% vs. 92%, *p*=0.01). Furthermore, the CRS subgroup analysis revealed that CAR-T cell therapy with 4-1BB in the CAR construct attained a lower risk of CRS than other costimulatory molecules (95% vs. 99%, *p*=0.05).

**Table 4:** Subgroup analysis results of ORR and CRS rate.

Subgroups	Overall response rate			Cytokine-release syndrome rate		
	No. of trials	ORR (95% CI)	P for difference	No. of trials	CRS (95% CI)	P for difference
**Mean age (years)**			0.02			0.55
≥55	9	0.92(0.87,0.97)		10	0.98(0.96,1.00)	
<55	2	0.99(0.96,1.02)		2	0.99(0.96,1.02)	
**Sex**			0.16			0.75
male ≥50%	6	0.92(0.85,0.99)		6	0.99(0.97,1.00)	
male <50%	4	0.97(0.95,1.00)		4	0.98(0.95,1.01)	
**CAR-T infusion dose**			0.07			0.38
high dose group(≥500 × 10^6 cells or 10 ×10^6 cells/kg)	7	0.98(0.95,1.00)		7	0.99(0.97,1.01)	
low dose group(<500 × 10^6 cells or 10 ×10^6 cells/kg)	3	0.93(0.89,0.98)		4	0.97(0.95,1.00)	
**CAR-T infusion time**			0.5			0.2
same day	5	0.93(0.90,0.97)		5	0.97(0.95,0.99)	
not same day	5	0.98(0.96,1.01)		6	0.99(0.97,1.01)	
**Antigen-recognition domain origin (BCMA)**			0.47			0.57
Human	3	0.94(0.87,1.00)		3	0.98(0.93,1.02)	
Murine	3	0.96(0.92,1.00)		4	0.96(094,1.01)	
**Costimulatory molecule**			0.16			0.05
4-1BB	5	0.95(0.92,0.98)		6	0.95(0.92,0.98)	
others	4	0.98(0.95,1.01)		4	0.99(0.97,1.01)	
**Median time from diagnosis (years)**			0.22			0.93
≥2	2	0.93(0.88,0.98)		2	0.98(0.96,1.01)	
<2	2	0.87(0.79,0.95)		2	0.98(0.95,1.02)	
**Lines of prior treatment**			0.78			0.94
≥4	4	0.92(0.88,0.96)		4	0.98(0.96,1.00)	
<4	4	0.92(0.87,0.99)		4	0.98(0.95,1.01)	
**Prior ASCT (%)**			0.03			0.41
≥50	4	0.99(0.96,1.02)		4	0.99(0.97,1.01)	
<50	5	0.91(0.84,0.97)		5	0.97(0.95,1.00)	
**ISS3 (%)**			0.0002			0.84
≥50	2	0.70(0.57,0.83)		2	0.99(0.95,1.02)	
<50	6	0.96(0.93,0.99)		6	0.98(0.97,1.00)	
**High-risk cytogenetics (%)**			0.25			0.99
≥50	3	0.97(0.92,1.02)		3	0.98(0.94,1.02)	
<50	3	0.93(0.89,0.98)		3	0.98(0.96,1.00)	
**Extramedullary Disease (%)**			0.05			0.89
≥20	4	0.87(0.78,0.97)		4	0.99(0.97,1.00)	
<20	1	0.99(0.93,1.05)		1	0.99(0.93,1.05)	
**mAb exposed (%)**			0.08			0.33
≥20	3	0.93(0.87,0.98)		4	0.95(0.92,0.98)	
<20	2	0.98(0.95,1.02)		2	0.99(0.95,1.02)	
**Conditioning treatment**			0.01			0.25
Cy+Flu	9	0.92(0.89,0.95)		9	0.98(0.96,1.00)	
Cy+Busulfan	3	0.99(0.96,1.02)		3	0.99(0.96,1.02)	

## Discussion

Modern advancements in MM treatment have progressed from conventional chemotherapies to more specific immune-based treatments. As a state-of-the-art method, anti-BCMA CAR-T cell therapies have attained remarkable success in RRMM. BCMA, mainly expressed in malignant plasma cells and multiple B cells, is viewed as an eligible target for RRMM CAR-T cell therapies (27). Previous studies have demonstrated that over 81% RRMM respond to anti-BCMA CAR-T cell therapies, however, most responsive patients tend to relapse eventually (6, 7, 27). Bruno et al. found that BCMA expression varied greatly among different MM patients, and that nonresponsive MM patients had plasma cells with lower expression of BCMA (28). The downregulation of BCMA after infusion of anti-BCMA CAR-T cell therapies, aligning with the onset of MM disease progression, was also observed (28, 29). These findings demonstrate that BCMA escapes the existence and insufficiency of solo antigen-targeting CAR-T cell therapy in MM treatments. The role of CD19 in B-cell lineage differentiation is significant, and its expression generally decreases in MM cells (30). Previous studies have demonstrated that a small population of less-terminally differentiated CD19^+^ plasma cells may construct a drug-resistant, clonogenic disease reservoir that is maintained by components of the bone marrow microenvironment (31, 32). These lead to consider CD19 as a promising target alongside anti-BCMA CAR-T cell therapies. This meta-analysis of 12 clinical trials, inclusive of 347 participants, indicates that combined anti-BCMA and anti-CD19 CAR-T cell therapy deliver remarkable benefits with a controllable safety profile in RRMM.

This meta-analysis, focusing on the efficacy and safety of combined anti-BCMA and anti-CD19 CAR-T cell therapy in RRMM, highlights that all clinical trials show substantial effectiveness. The pooled ORR was 94%, with the highest response rate of 100% reported by Xiaolan Shi et al. (23–25), and other results hovered around 90%, demonstrating the considerable effectiveness of the combined CAR-T cell therapy in RRMM. ORR only reflects the quantitative indicators of therapeutic response, while PR, VGPR, CR, sCR and other indicators can reflect the quality of remission. Therefore, we further analyzed the incidence of the therapeutic effect of CR+sCR and identified that the CRR was 50%. Due to the low rate of CR obtained by conventional treatments, even autologous transplantation could only achieve a CR rate of 40% (33), hence these results suggest significant efficacy in MM treatment using combined anti-BCMA and anti-CD19 CAR-T cell therapy.

In terms of effectiveness, compared with single-target CAR-T therapies, the combined anti-BCMA and anti-CD19 CAR-T therapy showed better efficacy. The reported ORRs were 77% by Yang Q et al. (34), 85.2% by Zhang L et al. (35), and 87% by Hu D et al. (36), which are all lower than our result (94%). Moreover, the reported CRRs were 37% by Yang Q et al. (34), 47% by Zhang L et al. (35), and 44% by Hu D et al. (36), which are all lower than our result (50%).

Despite the excellent efficacy, toxicities of CAR-T cell therapy in RRMM after treatment are also important. CRS, a systemic inflammatory response, is the most common AE following CAR-T infusion (37). It can result in clinical symptoms such as neurotoxicity, fever and hypotension (38). In this study, the overall incidence of any grade CRS was 98%, primarily of CRS grade 1 or 2. In addition, neurotoxicity is also a common adverse reaction. Neurotoxicity is a complex syndrome, including encephalopathy, cognitive defect, dysphasia, seizure and cerebral edema (39), which is thought to be one of the primary challenges to the general application of CAR-T cell therapy (40). The overall incidence of grade 3 or 4 neurotoxicity was low (8%). Generally, considering the high efficiency of combined anti-BCMA and anti-CD19 CAR-T cell therapies, the safety profile was manageable and tolerable. In the future, a more mechanistic understanding of AE is important to raise the efficacy-to-toxicity ratios of combined CAR-T cell therapies.

In terms of safety, the AEs of CAR-T cell infusion mostly presented as CRS and neurotoxicity (41). The reported incidences of any grade CRS were 82% by Hu D et al. (36) and 80.3% by Roex G et al. (5), which is lower than our result (98%). Otherwise, compared to single-target CAR-T therapy, there was little difference in the incidence of other AEs. The reported incidences of grade 3 or higher CRS were 14% by Yang Q et al. (34), 11% by Hu D et al. (36), 14.1% by Roex G et al. (35), which is all higher than our result (9%). The reported incidences of neurotoxicity were 10% by Hu D et al. (36), 10.5% by Roex G et al. (5) and 8% by Xiang, X (42), which is slightly higher than our result (8%).

The results of subgroup analysis provide valuable insights for the selection of RRMM patients suitable for the combined anti-BCMA and anti-CD19 CAR-T cell therapy. Subgroup analysis of ORR by characteristics of the included participants showed that MM patients with a better disease status tended to acquire better efficacy. Specifically, patients under 55 years old can obtain a higher ORR compared to those aged 55 years and above. Similarly, a greater ORR was observed in patients who had received ≥50% of prior ASCT, compared to those who received less than 50%. This could be attributed to the sequential treatment of ASCT followed by CAR-T therapy, as reported by Xiaolan Shi et al. (23–25), presumably placing patients in a good basic condition when starting the CAR-T treatment, thus enhancing the remission effect. Also, compared to the proportion with extramedullary disease ≥20%, a higher ORR was observed with the proportion <20%, probably because myeloma cells proliferate and accumulate in other organs, leading to a worse survival state and a poorer response to the therapy. Another insightful subgroup analysis was performed based on the percentage of ISS III in patients, showing a greater ORR for ISS III <50% as opposed to ISS III ≥50%.

The variations in costimulatory molecules may also have an impact on safety which is reflected in the incidence of CRS. The CRS subgroup analysis revealed that CAR-T cell therapy with 4-1BB in the CAR construct attained a lower risk of CRS than other costimulatory molecules. The second-generation CAR-T is created by combining the cytoplasmic domain of the CD3ξ chain with the signaling domains of co-stimulatory receptors, such as CD28 and 4-1BB, to give T cells a high potential for proliferation, activity, and persistence (43, 44). It has been established that costimulatory molecules play a crucial role in affecting T cell activation, proliferation, survival and cytokine release which regulate tumor immunity (45). Studies have shown that in preclinical studies, CARs with 4-1BB costimulatory domains typically lead to a relatively gradual and long-lasting response (46) and produce less cytokine release compared (47) with CD28, which may explain the smaller incidence of CRS in 4-1BB than other costimulatory molecules in our subgroup analysis. We also performed a subgroup analysis of ORR based on the antigen recognition domain origin of the CAR. The findings revealed that the effectiveness was similar in the Human group and Murine group, with no statistically significant difference, indicating that the species origin of the CAR antigen-recognition domain is not a major determinant of ORR of dual-target CAR-T therapy. However, Hu D et al. (2023) reported that humanized CAR-T cells were superior to those produced from murine as there are inherent limitations in the use of murine scFv-based CARs (36). Consequently, additional clinical studies are required to address this controversy.

In the subgroup analysis of conditioning treatment, nine of the trials were treated with Flu + Cy and three trials were treated with Busulfan + Cy. The results showed better efficacy in the Busulfan + Cy group. This may be due to Busulfan + Cy not only having myelosuppressive effects but also being anti-tumor to some extent (48), so using this as conditioning treatment before combined CAR-T cells infusion may have better efficacy. To explore whether infusion time would affect the results, we conducted a subgroup study. Among the 12 trials, except for one that did not mention the infusion time, five trials received BCMA CAR-T cells and CD19 CAR-T cells on the same day (three trials were infused in one day, and two trials were infused in three days). The remaining six trials received CD19 CAR-T cells on Day 0, 40% BCMA CAR-T cells given on Day 1, and 60% BCMA CAR-T cells given on Day 2. Although CAR-T cells were not infused on the same day, they all completed the infusion within a short period. The results showed that the difference caused by infusion time was not statistically significant.

## Conclusion

In summary, although considerable toxicity was observed, the combined anti-BCMA and anti-CD19 CAR-T therapy has a significant effect on the treatment for RRMM. Subgroup analysis revealed possible factors influencing efficacy and safety. These findings may inform the development of next-generation combined CAR-T cell therapy and the optimization of their clinical applications. Further, more well-designed clinical trials with large sample sizes are required to establish the role of combined CAR-T therapy in RRMM patients.

## Data availability statement

The original contributions presented in the study are included in the article/**Supplementary Material**. Further inquiries can be directed to the corresponding authors.

## Author contributions

HX: Conceptualization, Data curation, Formal analysis, Funding acquisition, Investigation, Methodology, Project administration, Resources, Software, Supervision, Validation, Visualization, Writing – original draft, Writing – review & editing. CG: Conceptualization, Project administration, Resources, Supervision, Writing – review & editing. PX: Conceptualization, Data curation, Software, Writing – review & editing. DZ: Project administration, Resources, Writing – review & editing. YX: Funding acquisition, Writing – review & editing. BC: Funding acquisition, Software, Writing – review & editing. HB: Conceptualization, Data curation, Formal analysis, Funding acquisition, Investigation, Methodology, Project administration, Resources, Software, Supervision, Validation, Visualization, Writing – original draft, Writing – review & editing.
