# Supplementary figures and images for: Clinical efficacy and safety of combined anti-BCMA and anti-CD19 CAR-T cell therapy for relapsed/refractory multiple myeloma: a systematic review and meta-analysis
Source: Front Oncol. 2024 Apr 8;14:1355643. doi: 10.3389/fonc.2024.1355643 (PMC11033299; doi:10.3389/fonc.2024.1355643)

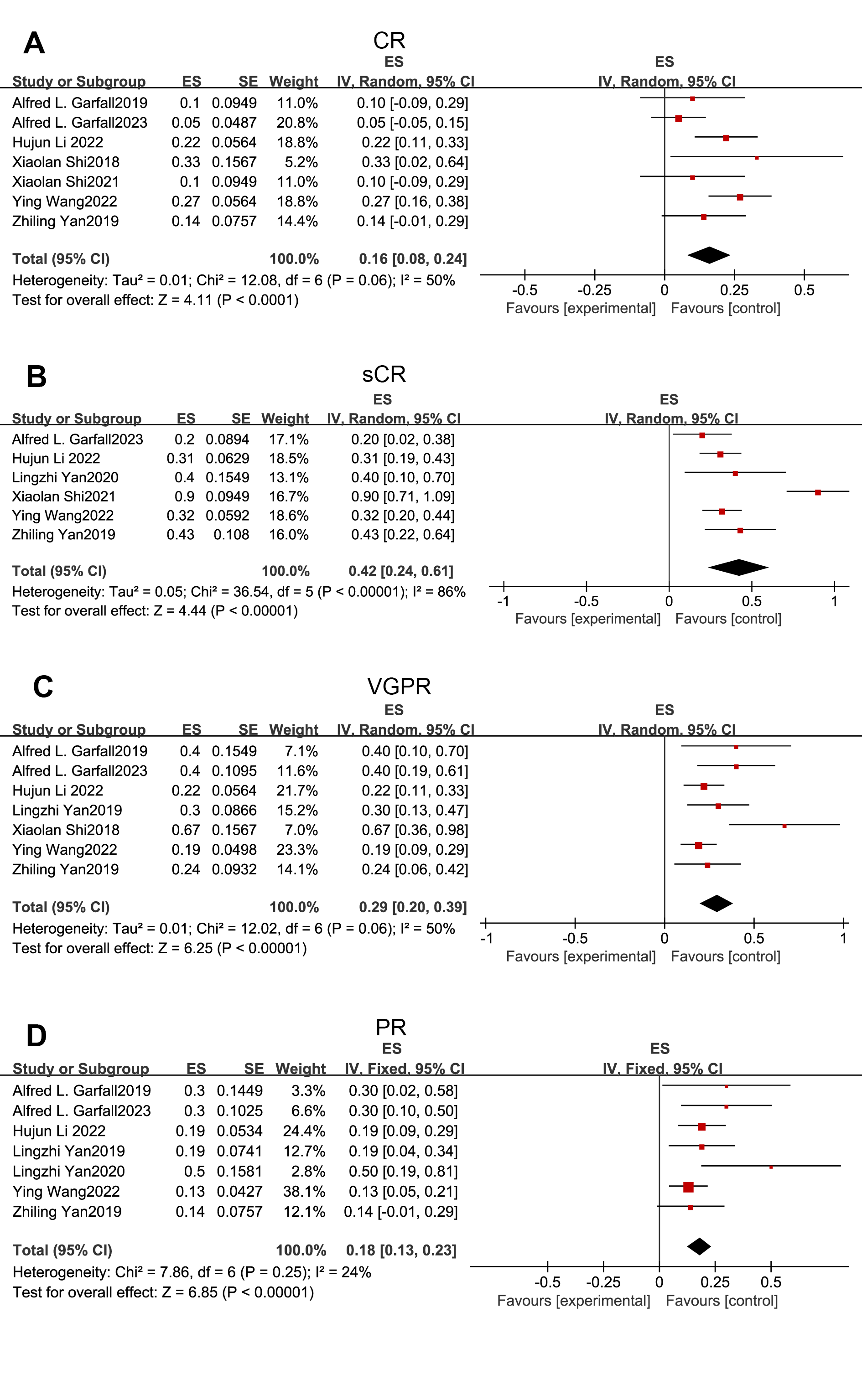

Supplement: Supplementary file 1 [file Image_1.tif]

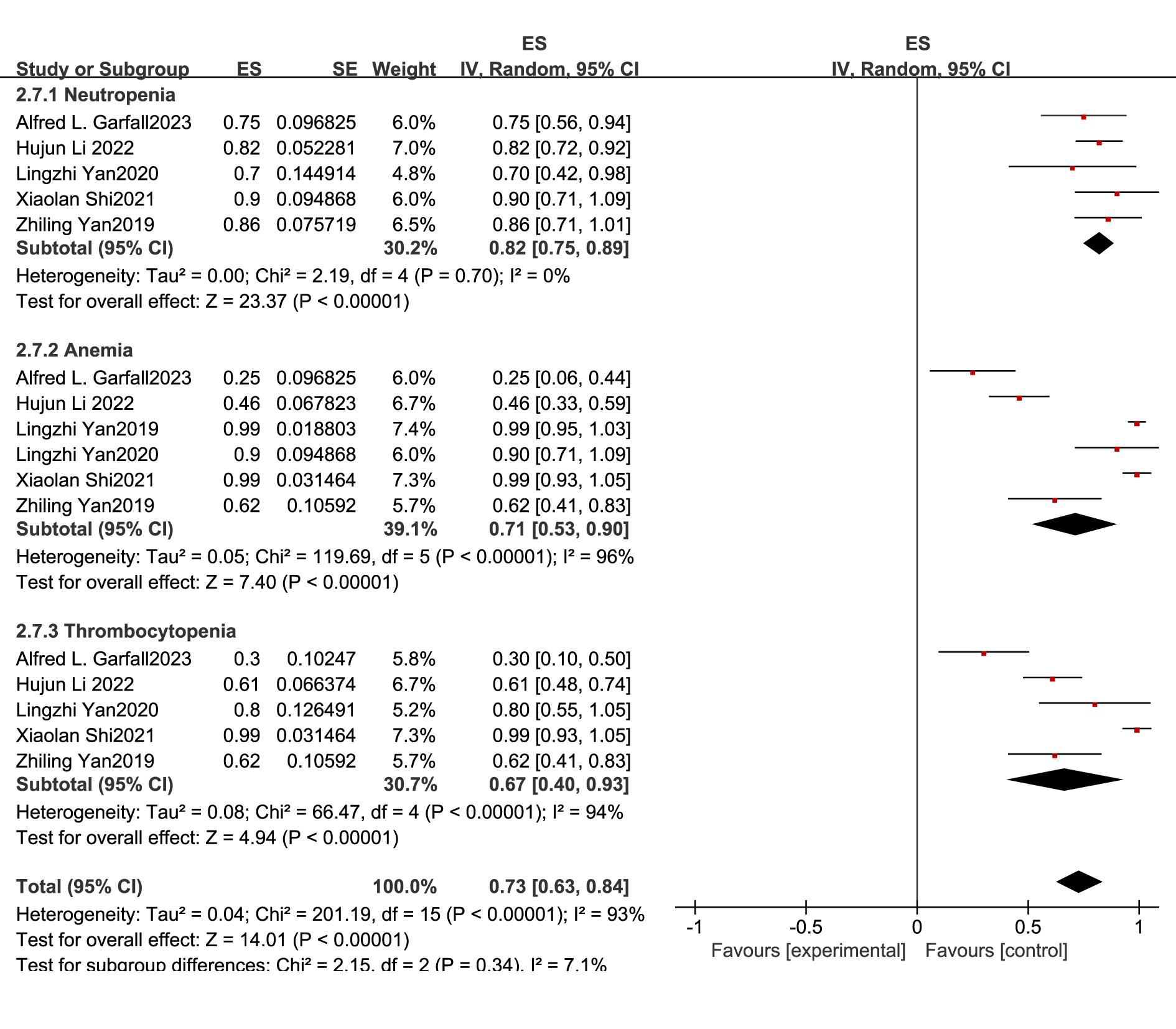

Supplement: Supplementary file 2 [file Image_2.tif]

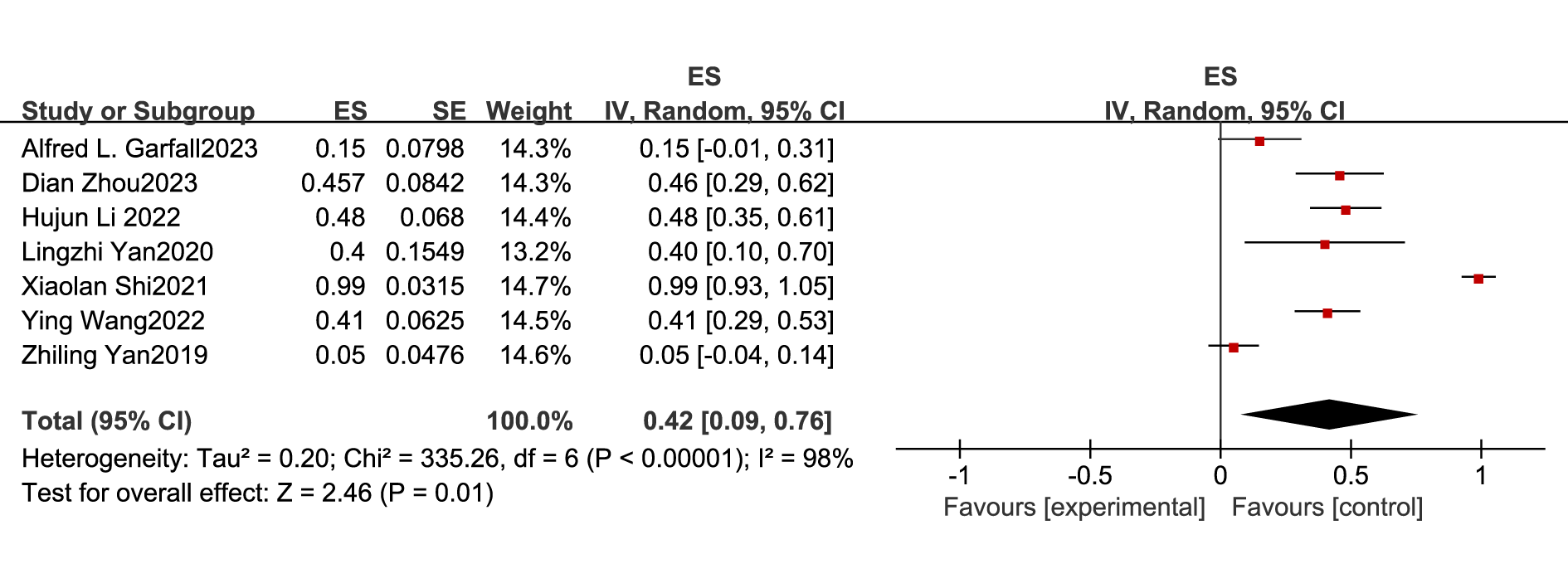

Supplement: Supplementary file 3 [file Image_3.tif]
